# Government funded suicide prevention in Australia – an environmental scan

**DOI:** 10.1186/s12889-024-19483-w

**Published:** 2024-08-26

**Authors:** Bridget Bassilios, Dianne Currier, Karolina Krysinska, David Dunt, Anna Machlin, Danielle Newton, Michelle Williamson, Jane Pirkis

**Affiliations:** Centre for Mental Health and Community Wellbeing, Melbourne School of Population and Global Health, University of Melbourne, Melbourne, Australia

**Keywords:** Suicide prevention, Suicide prevention policy, Suicide prevention services, Suicide prevention programs

## Abstract

**Background:**

Suicide is a worldwide public health problem. In response to this problem, Australia was one of the first countries to develop national suicide prevention policy. Guided by the National Suicide Prevention Office (NSPO), which was established in 2021, suicide prevention in Australia is in a period of reform. The NSPO is driving a nationally consistent and integrated approach to suicide prevention including leading the development of a new National Suicide Prevention Strategy. This article summarises findings from an environmental scan of government-led suicide prevention in Australia, conducted as an input for the development of the new Strategy.

**Methods:**

The scan was conducted from August 2022 to January 2023. We searched relevant government websites and Google to identify policy documents and programs and services. We undertook a desktop review of documents and programs/services using coding templates developed to address the objectives of the scan. Qualitative information was extracted in a systematic manner using these templates.

**Results:**

Australia’s suicide prevention efforts are significant as demonstrated by activities ranging from policy documents intended to guide and plan activity, the National Mental Health and Suicide Prevention Agreement committing the Federal Government and jurisdictions to work together, and the availability of national, state, local area based, and digital services and programs. Suicide prevention approaches in Australia are mostly selective or indicated. There is less emphasis on universal approaches, wellbeing promotion, strengthening protective factors and mitigating the impact of known drivers of distress. In addition, there is limited evidence to demonstrate a whole-of-government or whole-of-system approach is operating in Australia. Findings should be interpreted in the context that suicide prevention in Australia is currently in a period of transition.

**Conclusions:**

Current government emphasis on and investment in suicide prevention activity, together with strong commitment to lived experience and cross sectorial collaboration, are substantial and appropriate. There are also many opportunities to further progress cross-portfolio and cross-jurisdiction suicide prevention and response efforts. This requires urgently adopting a shared understanding of suicide, which includes the diverse drivers of suicidal distress, and improving protective factors and social wellbeing.

## Background

Suicide remains a pressing but preventable worldwide public health problem. Globally, around 703,000, people die by suicide each year [1]. Effective strategies for preventing suicide include training general practitioners to recognise and treat depression, routine active follow-up of patients after discharge or a suicide-related crisis, means restriction, and educating young people about depression and suicidal behaviour [2, 3]. Combination approaches in health care systems show promise in reducing suicide in several countries, but evidence is yet to be amassed [2, 3]. The World Health Organization endorses four evidence-based suicide prevention interventions including means restriction; interacting with media for responsible reporting of suicide; fostering socio-emotional life skills in adolescents; and early identification, assessment, management and follow up of anyone affected by suicidal behaviours [4].

In response to the problem of suicide, three decades ago, Australia was one of the first countries to have commenced developing and supporting national suicide prevention policy. However, the Australian suicide prevention rate is not decreasing, with 3,249 deaths by suicide recorded in 2022, representing an age-standardised rate of 12.3 per 100,000 people [5]. Furthermore, in Australia, suicide and self-inflicted injuries is the second leading cause of premature death from injury or disease and is the leading cause of premature death in men aged 15–49 years [6].

More recently, in 2019, the Federal Government announced the appointment of the first National Suicide Prevention Adviser to work with the National Suicide Prevention Taskforce to advise on reforming the suicide prevention system. The *National Suicide Prevention Adviser’s Final Advice (Final Advice)* drew heavily on the views of people with lived experience of suicide, in addition to government, service provider organisations and leaders in Indigenous suicide prevention [7–10]. The *Final Advice* is founded on ‘whole of system, whole of life’ principles, aiming to provide early intervention anywhere it could be needed in the service delivery system. This tenet is reliant on a whole-of-government approach to suicide prevention, which includes all government levels (Federal, state and territory, and local government) and all portfolios (not just health or mental health) working together on integrated policies and programs to prevent suicide and self-harm [7].

In 2021, the Federal Government announced the establishment of the National Suicide Prevention Office (NSPO) tasked with working across governments, portfolios, and sectors to drive the development of a nationally consistent and integrated approach to suicide prevention. This includes leading the development of a new National Suicide Prevention Strategy (Strategy), which will be followed by the development of a National Outcomes Framework and a National Suicide Prevention Workforce Strategy in 2024 [11].

Based on the blueprint for a public health, whole-of-government approach to suicide prevention developed by Pirkis and colleagues [12, 13], the *Final Advice* [7–10] and a range of other inputs, the NSPO has been iteratively developing a framework for the new Strategy structured around focus areas and enablers (Table 1) [14]. The focus areas are the critical domains where action is required by governments and service providers to significantly reduce suicide and suicidality. The enablers are foundational areas of system reform required to drive the effective implementation of the Strategy as well as strengthen suicide prevention efforts more broadly. The enablers reflect some of the World Health Organization’s endorsed necessary pillars for implementing suicide prevention in countries such as multisectoral collaboration; capacity building; and surveillance, monitoring and evaluation [4].

**Table 1:** NSPO framework for the development of the new National Suicide Prevention Strategy as at 2022 [14]

Concept	Description
Focus areas	• Strengthening protective factors and wellbeing • Mitigating the impact of known drivers of distress • Empowering earlier intervention • Providing accessible, comprehensive, and compassionate care • Supporting long-term wellbeing
Enablers	• Governance and collaboration across governments and portfolios • Embedding lived experience decision-making and leadership • Data and evaluation • Workforce and community capability

Since writing this article, the framework for the Strategy has been refined through consultation with stakeholders and will be modified by the time of publication. Therefore, although the Strategy’s refinements are consistent with the broad policy positions reflected and the findings in this article, readers should access the latest version of the Strategy

The NSPO commissioned the University of Melbourne’s Centre for Mental Health (now named the Centre for Mental Health and Community Wellbeing) to conduct an environmental scan of the government-led suicide prevention system in Australia as an input for the development of the Strategy. The scan aimed to address the following research questions:

1. What are governments in Australia (Federal and state/territory) doing to prevent suicide?
2. To what extent is government-led suicide prevention activity in Australia aligned with the focus areas in the NSPO framework for the new Strategy?
3. To what extent does government-led suicide prevention activity in Australia leverage the system enablers described in the NSPO framework for the new Strategy?

This study reports the findings of this environmental scan, which may be used to inform the development of national approaches to suicide prevention in other high-income countries or to compare approaches between countries.

## Method

### Data sources and scope

The scan was conducted from August 2022 to January 2023 and focused on two key methods: (1) a desktop review of government policy and agreement documents and (2) a scan of government-led/or directly funded programs and services. The former included Federal and state/territory documents and the latter focused on national programs and services. Supplementary key informant interviews were also conducted and will be reported separately. The scope of the environmental scan focused on government led activity because its findings will inform the development of the new National Suicide Prevention Strategy, which is a government-led document.

#### Government policy and agreement documents

We identified the current or most recent publicly available Australian suicide prevention ‘policy documents’ (e.g., government agreements, strategies and plans) by referring to documents mentioned in the *National Suicide Prevention Adviser’s Final Advice* [7–10] and by using the search terms ‘suicide prevention policy’, ‘suicide prevention strategy’, ‘suicide prevention plan’, and ‘suicide prevention agreement’. These search terms were entered into the relevant websites including Federal, state and territory government; established suicide prevention organisations and agencies; Primary Health Networks (PHNs, which are funded by the Federal government and commission health services to meet needs of their local communities); and Google. The search was not restricted to health portfolios but included suicide prevention activities undertaken within any government portfolio or agency.

#### Programs and services scan

Because a single data source for programs and services was not found, we identified selected key programs and services in the national service system from similar websites used to identify policy documents, and contacted staff from relevant agencies to ensure that key initiatives about which information was not in the public domain were included in the scan. Only programs or services labelled, funded, or designed explicitly with the objective of suicide prevention were included.

### Data extraction and analysis

We undertook a desktop review of documents collected. We developed coding templates based on the aims and objectives of the scan and on our discussions with the NSPO to summarise and organise salient themes as they emerged from the given data source. Separate coding templates were developed for the different focus areas and enablers, but we attempted to align the coding frameworks across different data sources. For example, data extracted from policy documents included: Title of policy document, Period, Who is involved, Roles and responsibilities, Key objectives, Approach/guiding principles/conceptual framework, Priority populations, Interventions/programs, Funding/budget, Monitoring/evaluation/outcome measurement, and Evaluation of plan’s progress in relation to suicide prevention activity. Data were also extracted from these documents on lived experience and workforce.

Qualitative information was extracted in a systematic manner using these templates. This analysis enabled us to describe areas of commonality and difference across policy documents. Having described each policy or program, we considered the extent to which their totality demonstrates progress within a particular focus area or enabler of the NSPO framework for the new Strategy. Progress was assessed as considerable, partial or mixed, absent, or not possible to assess.

## Findings

Table 2 outlines the number and type of documents reviewed and data extracted. Appendix 1 provides a detailed list of the documents.

**Table 2:** Key policy documents included in scan and data extracted

Document type	Data extracted	Number of documents
Federal and state/territory plans, strategies, and frameworks (policy documents)	• Alignment with all focus areas and enablers of the NSPO framework • Identification of programs and services	19
National Mental Health and Suicide Prevention Agreement (*National Agreement*) and bilateral agreements with each state and territory	• Alignment with all focus areas and enablers of the NSPO framework • Identification of programs and services	9
Additional policy documents and reports from inquiries or reviews	• Alignment with workforce and lived experience elements of the NSPO framework	13
Joint regional plans for integrated mental health and suicide prevention services covering 30 PHN regions	• Alignment with all focus areas and enablers of the NSPO framework • Identification of programs and services	28

*PHN* Primary Health Network

Findings are presented for each research question. Space does not permit a complete listing of activities, programs and services, so key examples are presented.

### Research question 1. What are governments in Australia doing to prevent suicide?

Led by Federal and state and territory governments, Australia’s suicide prevention and response efforts are extensive across five activity categories: (1) Strategies, plans, and frameworks, (2) the National Agreement, (3) Joint regional plans, (4) Key programs and services, and (5) Monitoring and evaluation.

#### Suicide prevention and response in policy documents (strategies, plans and frameworks)

Many Federal and state and territory strategies, plans and frameworks are in place, focussing explicitly on suicide prevention or considering it together with mental health. Most strategies include a consideration of priority populations and standalone national priority population suicide prevention strategies exist for First Nations peoples [15] and LGBTIQ + people, although government contributed to rather than developed the latter strategy [16, 17]. Federal mental health and wellbeing strategies for current and ex-Australian Defence Force (ADF) members [18, 19] and children [20] include suicide prevention.

The Zero Suicide Framework, which aims to improve support for people in crisis presenting to emergency departments, was mentioned as a cross-portfolio initiative in several policy documents. For example, to improve data and evidence, the national Veteran Strategy mentions a formal monitoring and evaluation plan that will align with the Government’s Towards Zero Suicides agenda [19]. Queensland and New South Wales have published care pathways based on the Zero Suicide Framework [21, 22]. The Zero Suicide Framework is being implemented in Queensland to drive cultural and clinical change in suicide care across all hospital and health services [23], and in New South Wales to trial evidence-based peer support and peer-led initiatives as alternatives for people with suicidal ideation presenting to emergency departments [24]. The South Australian plan also includes the Zero Suicide Framework [25].

#### National Mental Health and Suicide Prevention Agreement (*National Agreement*)

The *National Agreement* [26] is a key mechanism for formalising a joint Federal-state/territory approach including Federally funded PHNs and Medicare Benefits Schedule (MBS) services and state/territory government funded Local Hospital Networks (LHNs); as well as an explicit commitment to a whole-of-government approach to supporting and funding suicide prevention activity in portfolios other than health, e.g., education, justice, disability, housing etc.

Under the *National Agreement* [26], the Federal Government and state and territory governments are working together to support key initiatives including a Distress Brief Intervention (DBI) Trial, postvention services, and the national rollout of aftercare services for people following a suicide attempt (universal aftercare^1^). Table 3 briefly describes these initiatives.

**Table 3:** Key suicide prevention and response initiatives under the National Agreement

Initiative	Description	Funding
Distress Brief Intervention Trial	Under development in partnership with people with lived experience of emotional distress based on a model piloted in Scotland [27]. Training for non-clinical frontline staff in community settings where people are not seeking help for their distress (e.g., social services) to provide a compassionate response (Level 1) involving referral for 14 days’ intensive support provided by trained community sector staff (Level 2). Includes developing personalised distress management plan and improving coordination and integration between services.	Co-funded by Federal and state governments in New South Wales, Victoria, and Queensland where it will be trialled
Postvention	Free face-to-face and/or telephone support (including connections to local services) offered seven days per week for individuals, families, communities and first responders bereaved by suicide; and available in all states/territories. Over three years, selected states/territories will trial enhancing standard postvention services through suicide bereavement counselling and peer support.	Co-funded by Federal and state/territory governments in New South Wales, Victoria, Queensland, and Northern Territory Federally funded in the other states/territories
Universal aftercare services	Up to three months’ follow-up non-clinical support and coordination of care for all people who have attempted suicide or experienced a suicidal crisis. The care is provided by trained support coordinators with flexibility for states/territories to select universal aftercare service delivery models to meet local community needs. Universal aftercare services will trial expanded referral pathways beyond hospital settings to support people experiencing suicidal crisis who do not present to hospital.	Co-funded by Federal and all state/territory governments except South Australia Federally funded in South Australia

#### Joint regional plans for integrated mental health and suicide prevention

Under Australia’s *Fifth National Mental Health and Suicide Prevention Plan (Fifth Plan)* [28], Federal, state and territory governments require PHNs and LHNs to jointly develop plans for integrated mental health and suicide prevention services in their local regions (geographic locations). These joint plans aim to address local service-based problems faced by people with lived experience of mental illness or suicide and their carers and families, such as fragmentation, gaps, duplication and inefficiencies in service provision, and a lack of person-centred care [29]. Joint regional plans have been developed for all of Australia’s 31 PHN locations (one plan was published after our scan was completed, so is not included in this analysis). Plans cover periods of 2–5 years; some are foundational, and others outline activities and outcomes regarding strategic priorities. The Federal Government funds PHNs and joint local area-based regional planning and state and territory governments fund LHNs.

#### Key programs and services

Australia’s suicide prevention service system, comprising government agencies, service providers and the non-government sector, is complex [30]. This section describes key initiatives other than those available through the MBS, from hospitals or under the *National Agreement* including: (1) the National Suicide Prevention Leadership and Support Program (NSPLSP), (2) PHN-commissioned services, (3) national digital mental health and suicide prevention services and programs, and (4) online navigation systems.

##### National Suicide Prevention Leadership and Support Program (NSPLSP)

The NSPLSP was introduced in 2017 as part of the Federal Government’s response [31] to the *National Review of Mental Health Programs and Services* [32] and is key among national suicide prevention efforts. The program is a mechanism for providing essential sector leadership, reform, advocacy, research and translation, and services targeting people who are disproportionately impacted by suicide. Currently, the Federal Government funds 40 projects via the NSPLSP to perform seven suicide prevention activity category types (described in Appendix 2): (1) National leadership in suicide prevention; (2) National leadership in suicide prevention research translation; (3) Centre of Best Practice in Aboriginal and Torres Strait Islander Suicide Prevention (CBATSISP); (4) National support for lived experience of suicide; (5) National media and communications strategies; (6) National suicide prevention training; and (7) National suicide prevention support for at risk populations and communities.

Most NSPLSP projects provide selective interventions targeting specific at-risk populations (e.g., youth, men experiencing psychosocial distress, people bereaved by suicide, people in rural/remote locations, veterans, LGBTIQ +, and First Nations peoples); or people/organisations supporting them (e.g., PHNs). The NSPLSP also funds the CBATSISP.

The NSPLSP is currently being evaluated by Australian Healthcare Associates. Projects funded under the NSPLSP are evidence-informed [33] meaning they are based on a combination of research evidence, lived experience views, professional expertise, and information from the practice context [34]. An earlier evaluation of the NSPLSP found that although there were reporting inconsistencies, most projects were achieving their targets and had the potential to strengthen or expand their activities through additional funding [35]. It also found that a definition of suicide prevention leadership in the context of the NSPLSP was lacking, engagement (and associated outcomes) between PHNs and NSPLSP projects was mixed, project leaders were unaware of each other’s activities, and many activities were universal or generalist [35].

##### PHN-commissioned suicide prevention services and activities

PHNs are Federally funded organisations that coordinate primary health care in their local region. PHNs receive government funding to commission suicide prevention and mental health services including those covered in the *National Agreement* [26] such as universal aftercare. Other services commissioned by PHNs range from low or moderate to higher intensity stepped mental health care matched to consumer level of need or symptom severity (e.g., low intensity digital mental health and suicide prevention services, primary mental health care for people with moderate symptoms/need, higher intensity in-person services for people with severe and complex symptoms/needs, respectively). Additionally, all 31 PHNs have received funding to appoint a Suicide Prevention Regional Response Leader to work with communities in their catchment to determine local needs and target programs and services commissioned [17].

Many PHNs participated in multi-component program suicide prevention trials between 2017 and 2022; the evaluation findings from which have influenced policy directions [36, 37]. For example, the National Suicide Prevention Trial showed that four of 12 Trial Sites (11 PHNs) commissioned aftercare services for people who had attempted suicide or were experiencing a suicidal crisis, and all Trial sites commissioned a range of community-based activities including some led by non-government organisations [36]. Community-based activities mostly involved either awareness raising and engagement activities or capacity building, such as providing training to community members, frontline workers and members of the health and allied health workforce on suicide prevention or offering mental health first aid training.

##### National digital mental health and suicide prevention services and programs

Digital services are delivered remotely via telephone, videoconference, online chat, online course (self- or therapist-guided), secure mobile messaging (SMS) or mobile applications (apps). Digital mental health services have been funded by the Federal Government since 2006. Twenty digital mental health and suicide prevention services were reviewed as part of this scan irrespective of funding source either because they are suicide prevention or postvention specific, offer mental health support including the ability to manage suicidal distress, or target at-risk populations. Appendix 3 provides an overview of the characteristics of these 20 services.

Most of the 20 digital services reviewed provide selective (e.g., young people, First Nations peoples, people who identify as LGBTIQ+, veterans) or indicated interventions for anyone experiencing distress (including families and those bereaved through suicide), crises and mental health or other problems. Most are available 24 h per day and/or operate 365 days and/or offer extended hours, which means people can get the care they need when they need it and where they need it. This flexibility is facilitated by many digital services using multiple communication modalities (phone, online, mobile applications, email). Digital services are generally delivered by qualified counsellors or other mental health professionals, some are delivered by peers or trained volunteers, or a combination of options. Two services are delivered by First Nations peoples (13 YARN and National Indigenous Postvention Service).

Evaluation findings were located for 11 of the 20 digital services we reviewed. These evaluations show that digital services are valued by users, are effective (e.g., lead to improvements in wellbeing, increase help seeking, reduce suicidality), and/or contribute to service improvements.

##### Online navigation systems

To improve care coordination and integration and help people find the care they need, a range of service navigation systems have been created over time. Navigation – involving engagement, assessment, service identification, referral, and monitoring/follow-up – can be performed by staff or through online web-based applications [38]. Ten online navigation systems funded by the Federal Government (with or without additional funding sources) were identified and are described in Appendix 4. Five online navigation systems are intended for use by consumers (and providers) including Head to Health, its redeveloped form as the National Mental Health Platform, the CBPATSISP Clearing House, Healthdirect and ReachOut. Three navigations systems are devoted to priority populations – two to First Nations peoples (CBATSISP Clearing House, WellMob) and one to young people (Reach Out – Tools and apps). Only three are suicide-prevention specific – Life in Mind, Suicide Prevention Australia’s Best Practice Directory and the CBPATSISP Clearing House. Five are broader mental health navigation systems that include information regarding suicide prevention. A key challenge associated with maintaining service navigation systems is keeping them up to date.

Evaluation information was mentioned regarding seven of the navigation systems. For some, this involved conducting internal quality assurance and service improvement activities. Published evaluations were found for fewer navigation systems. For example, an independent evaluation of the Head to Health gateway reported it has been used by a substantial number of people and has potential to be cost effective, but needs to be more widely promoted and user experiences can be improved [39]. Multiple studies have examined HealthPathways with a published review reporting that awareness and use are the most reported [40]. This review also reported that the impacts and outcomes of HealthPathways are difficult to measure due to “limitations in primary data and the interconnectedness of change” and called for “specific methodologies sensitive enough to capture the impact …over time” [40], which is applicable to evaluating navigation systems more broadly.

#### Monitoring, evaluation and research activity

Federal and state and territory governments monitor and evaluate policies, programs and services, and fund research to strengthen the evidence base. Examples include funding the Australian Institute of Health and Welfare (AIHW) to conduct the National Suicide and Self Harm Monitoring Project [41], and the suicide prevention trials delivered through PHNs and their evaluations [36, 37].

Additionally, the government commissions independent reviews and inquiries to help identify system problems, gaps, and opportunities for improvement. Examples include the *National Suicide Prevention Adviser’s Final Advice* [7–10], the *Productivity Commission’s Mental Health Inquiry* [42], the *Royal Commission into Victoria’s Mental Health System* [43], and the *National Review of Mental Health Programmes and Services* [32]. However, evaluation efforts including ensuring evaluation reports are available in the public domain need to be more consistent to ensure that implementation and outcome lessons are shared, effective approaches are sustained, and inadequate approaches are improved.

### Research question 2. To what extent is government-led suicide prevention activity in Australia aligned with the focus areas in the NSPO framework for the new Strategy?

The scan identified that there is currently only partial progress in aligning government-led suicide prevention activity in Australia with the five focus areas in the NSPO framework for the new Strategy including: (1) strengthening protective factors and wellbeing; (2) mitigating the impact of known drivers of distress; (3) empowering earlier intervention; (4) providing accessible, comprehensive, and compassionate care; and (5) supporting long-term wellbeing.

#### Focus area 1: strengthening protective factors and wellbeing

The *Pandemic Response* strategy was the only nationally coordinated government-led plan included in the scan that explicitly focussed on strengthening protective factors [44]. It included actions from the Federal and state and territory governments and multiple portfolios including those responsible for housing, employment and income support.

In terms of other policy and system responses aimed at strengthening protective factors and wellbeing under the remit of government-led suicide prevention activity, the emphasis on a whole-of-government approach in the suicide prevention policy documents included in the scan supported the principle of a comprehensive, coordinated policy and system response. Moreover, all the policy documents reviewed proposed multi-component approaches that included interventions aimed at strengthening protective factors and wellbeing such as early intervention, adopting a strengths-based approach, and building resilience. Strategies or elements of strategies focussed on First Nations suicide prevention foregrounded social and emotional wellbeing and cultural strengthening. In practice, most interventions named in strategies and agreements recognise and target individual risk factors (e.g., individual socio-demographic and contextual risk factors) [12].

Funded services and interventions reviewed in the scan, likewise, showed a predominantly risk-factor focus, with programs in educational settings being the most likely to focus on protective factors and general mental health and wellbeing. For example, the national initiative, Be You, is delivered to the entire school community and aims to promote and protect positive mental health in children and young people, although it also addresses risk factors such as bullying and the need for postvention [45]. Other services/interventions that address protective factors such as interpersonal and community connection and resilience are generally only selectively applied, for example, to men as an at-risk group, young people, or First Nations-specific cultural strengthening. Thus, although policy documents acknowledge a need for a population-wide approach across a broad range of psychosocial and socioeconomic domains as part of a comprehensive approach to suicide prevention, the range of interventions proposed as well as the current service/intervention landscape in the government-led suicide-specific domain are almost entirely risk-factor focussed and includes little activity at a population-wide level aimed at strengthening protective factors and wellbeing in general.

#### Focus area 2: mitigating the impact of known drivers of distress

All the policy documents reviewed in the scan acknowledged social determinants as contributing to suicide risk, and their adoption of the principle of a whole-of-government approach reflects an attempt to include social determinants as targets for intervention.

Individuals who are experiencing distress and/or suicidal crisis in the context of social determinant risk factors are mentioned in some strategies as high-risk populations and targeted for interventions (First Nations peoples, current and ex-ADF personnel, children and youth and LGBTIQ + people, residents in rural and remote regions, etc.). The main approach in terms of interventions for such individuals is to provide awareness and training across a diverse range of government agencies and services to recognise and potentially intervene to support those experiencing distress. The Queensland strategy is explicit in identifying every contact with a government agency as an opportunity for intervention [23]. However, the scan did not identify any government-led policies, services, or supports aimed at reducing the prevalence of distress in those contexts, or any system-wide measures to reduce the prevalence of those drivers.

The scan did not identify any strategy, service or intervention addressing upstream mitigation of social determinant-related drivers of distress although addressing distress in general as a risk factor for suicide is mentioned in a number of the bilateral schedules of the *National Agreement* [26]. Likewise, the majority of activities mentioned in the joint regional plans and included in the service system scan focused largely on proximal interventions to address suicide distress and crisis, or gatekeeper training to identify and support individuals experiencing any type of distress not necessarily related to social determinants.

There is likely substantial activity and services in place dealing with distress that fell outside the scope of the scan because they may be in non-health policy and service areas, and/or in the non-government sector.

#### Focus area 3: empowering earlier intervention during life transitions

Significant life transitions can increase vulnerability to suicidal distress. Examples include disengagement and transition from educational settings, leaving the defence force, release from correctional facilities, relationship breakdown and change in family structures, migration and settlement, bereavement, and change in work status due to unemployment, illness or injury. Policy documents acknowledge the importance of early intervention during life transitions to differing extents.

A range of suicide prevention activities contribute to progressing earlier intervention during life transitions, such as the previously described Distress Brief Intervention Trial and postvention services (see Research question 1). Other early intervention activities, some funded through the NSPLSP, include: therapies or services targeting non-suicidal distress; programs such as the Villy app for people transitioning to civilian life from the military; services and programs for young people such as headspace providing a range of early intervention services in clinical, educational and workplace settings, and YouthLife4life and Batyr delivering peer-led mental health and suicide prevention activities in educational settings; and some PHN-commissioned services that target certain populations experiencing difficult life transitions (e.g., people experiencing homelessness, refugees and asylum seekers, people in contact or at risk of contact with the justice system, and children with parents who have mental health problems).

Improving whole of population awareness of suicide prevention including how to provide or seek help during difficult life transitions may also help to foster earlier intervention. The NSPLSP funds eight projects aimed at awareness raising to reduce the stigma around suicide and encourage help seeking.

#### Focus area 4: providing accessible, comprehensive, and compassionate care

All the policy documents recognised the need for accessible and coordinated care. Although compassionate care was not referenced in all policy documents, related concepts such, ‘person-centred’ care were mentioned.

Overall, Australia’s suicide prevention services are largely affordable, with most, if not all, services being free of charge, which increases their likelihood of being accessible. The scan did not identify evidence for the comprehensiveness of the mental health and suicide prevention service system in terms of its capacity to respond to an individual’s unique co-occurring stressors across disciplines beyond mental health and health (e.g., financial, housing, legal, interpersonal, etc.).

Findings addressing Research question 1 described several initiatives intended to facilitate accessible, comprehensive and compassionate care, including digital services (by overcoming access barriers), navigation systems and PHNs through their knowledge of local services, the Distress Brief Intervention Trial, postvention, and aftercare for all people who have made a suicide attempt. Two additional key system components that have the potential to help improve access and navigation are Head to Health centres and peer-based service models, described below.

The Head to Health Centres and satellite network (previously Adult Mental Health Centres [AMHCs] and HeadtoHelp) are community-based adult mental health services delivered by multidisciplinary teams who provide holistic, collaborative care. These include eight new Head to Health Centres, 24 satellites embedded into existing primary care settings, the continuation of the initial eight AMHCs (one in each state and territory), and a central intake phone service [46]. The Initial Assessment and Referral Decision Support Tool (IAR-DST) is used to conduct central intake and is intended to improve accessibility, promote integration and facilitate referral to appropriate services [47]. Evaluation of HeadtoHelp shows that the service reduces psychological distress [48]. This evaluation also made recommendations for improving the effectiveness and consistency of the IAR-DST including increasing awareness and training in its use, supporting it with an up-to-date service directory and regular evaluation and review [48].

Additionally, a new network of 15 Head to Health Kids Hubs (mental health and wellbeing centres) for children aged 0–12 years is under development through the bilateral agreements under the *National Agreement* [26]. The Hubs aim to improve early intervention outcomes for children’s mental health and wellbeing by providing comprehensive, multidisciplinary care for children and their families [49]. This initiative builds on the findings of the *National Children’s Mental Health and Wellbeing Strategy* [20] and the Productivity Commission’s Mental Health Inquiry [42].

Peer-based services are increasingly emerging as non-clinical models that have potential to improve accessibility and provision of compassionate care [50, 51]. For example, around one third of the 18 projects funded under the NSPLSP’s National Suicide Prevention Support for At Risk Populations and Communities component involve peer-delivered service models (e.g., Safe Spaces as an alternative to emergency department).

To facilitate compassionate care, the Australian Public Service Mental Health and Suicide Prevention Unit has developed Compassionate Foundations: Suicide Prevention Capability Suite [52]. This is a self-directed, online foundational suicide prevention capability course to support positive interactions that promote connection and understanding.

#### Focus area 5: supporting long-term wellbeing

In principle, policy documents recognised the need for coordinated psychosocial support and integration of care for individuals experiencing a suicidal crisis and their families and carers. At the local state/territory and PHN level, work on developing care navigation and care pathways for people experiencing suicidal crisis to coordinate support is underway. However, mechanisms to deliver integration and coordination remain underdeveloped. The fragmentation of the mental health and suicide prevention service sector and workforce capacity and distribution impact on the ability to support ongoing, coordinated care models. Moreover, in terms of ongoing support for mental health and wellbeing, the current government-led suicide prevention landscape reflects the underlying relatively short-term funding cycles and the lifecycles of government policy and strategies which present a challenge to establishing a system of sustainable services and programs needed to support long-term recovery.

Because the scan specifically focused on suicide prevention policy and services, the majority of which respond to acute suicidality, few services were identified with capacity or service models designed to support longer-term, less acute distress through to a state of wellbeing.

### Research question 3. To what extent does government-led suicide prevention activity in Australia leverage the system enablers described in the NSPO framework for the new Strategy?

The scan identified that there is currently only partial progress in government-led suicide prevention activity in Australia leveraging the system enablers described in the NSPO framework for the new Strategy. This finding applies across all four system enablers including: (1) governance and collaboration across governments and portfolios; (2) embedding lived experience decision making and leadership; (3) data and evaluation; (4) workforce and community capability.

#### System enabler 1: governance and collaboration across governments and portfolios

Almost all the policy documents reference a commitment to a whole-of-government approach, particularly those which were more recently developed and those which explicitly drew on the *National Suicide Prevention Adviser’s Final Advice* [7–10]. However, details about how this approach would be operationalised was variable and limited. New South Wales and Queensland strategies offered more detail including which portfolio or agency have carriage of strategy elements [23, 24].

Given that many strategies are currently under, or will soon be due for, renewal there is an opportunity to progress coordination and integration of suicide prevention across and between jurisdictions. Most policy documents do not come with budgets attached, with some but not all jurisdictions providing implementation plans. Likewise, the short tenure of many suicide prevention strategies impacts on their ability to guide long-term structural changes that may be required to achieve a strong whole-of-government approach.

#### System enabler 2: embedding lived experience decision-making and leadership

Most of the policy documents (over 80%) and one third of joint regional plans specifically referenced people with lived experience and the process of co-design.

Lived experience was one of the specific target areas of the NSPLSP and lived experience leadership in suicide prevention, postvention, and peer support were cited as key achievements of the National Suicide Prevention Trial [53]. According to the *National Suicide Prevention Strategy for Australia’s Health System 2020–2023* [54], the term ‘evidence-informed’ encompasses four sources of evidence, including the qualitative insights of people with lived experience of suicide.

Specific actions related to lived experience at the national level were identified. For instance, the *Fifth Plan* [28] included involving consumers and carers in the Suicide Prevention Subcommittee reporting to Mental Health Drug and Alcohol Principal Committee and in the evaluation of the *Fifth Plan*. Further, to promote lived experience research, co-design and/or service delivery, the ALIVE National Centre for Mental Health Research has been established, the Roses in the Ocean CARE connect service (a peer operated suicide prevention call-back service) has been funded, and a collaboration has been formed between the Black Dog Institute (University of New South Wales) and the Aboriginal and Torres Strait Islander Lived Experience Centre.

At the state and territory level, policy documents mention plans to involve people with lived experience of suicide through co-design in redesign of mental health services, employment and membership in suicide prevention policy and governance, as well as development and trialling of peer support and peer-led initiatives.

#### System enabler 3: data and evaluation

The scan identified a range of issues related to suicide prevention and self-harm data and evaluation. Problems include concerns about the availability and quality of routinely collected data, inconsistent monitoring and evaluation, and limited evidence for effective interventions.

Most policy documents note the need for improved data and identify gaps in the current data. For example, there are inconsistencies in, and lack of recording of, suicide attempts across services (between jurisdictions, hospital vs. ambulance records, etc.) [54]. Additionally, problems applicable to broader health data also apply to suicide prevention and self-harm data, such as difficulty identifying priority populations including First Nations peoples, people who identify as LGBTIQ + and people from CALD backgrounds [8–10, 16].

Monitoring and evaluation of strategies and plans that guide suicide prevention activity is inconsistent. However, the National Mental Health Commission independently monitors and reports on the national mental health and suicide prevention system. Monitoring and evaluation of joint regional plans and the interventions, services, and programs they include is inconsistently specified. Many, but not all, Australian suicide prevention services and programs have been evaluated. Determining the effectiveness of all services and programs is important to increase our understanding and knowledge of what works to prevent, and respond to, suicide, and for government to make informed decisions about services and programs in which to invest.

The scan also identified activities that have been implemented to address some of the suicide prevention and self-harm data and evaluation problems identified. Key among these are the National Suicide and Self-harm Monitoring Project and the LIFEWAYS Project.

The Federal Government has funded the AIHW to conduct the National Suicide and Self-harm Monitoring Project from 2019–2020 to 2024–2025. The project has developed and is expanding a monitoring system to improve the quality, accessibility and timeliness of suicide and self-harm data in at-risk groups and regions in Australia [41]. The project intends to support: the development of effective policies, programs and interventions; the delivery of tailored services; reduction in suicide and self-harm rates; and tracking progress [55].

Funded under the NSPLSP, the LIFEWAYS Project provides capacity building of the suicide prevention research workforce and translation of research into policy and practice. Its research priorities study identified that existing research and evidence is heavily weighted toward risk factors, and future suicide prevention research should address suicide attempts, protective factors, social determinants, community settings, and interventions, and focus on strengthening effective research translation into practice [56]. These priorities represent areas requiring increased emphasis rather than the deprioritising of research with other focuses (e.g., epidemiology, suicide, suicidal ideation, suicide method, priority groups, etc.).

#### System enabler 4: workforce and community capability

Over 75% of the policy documents refer to ‘suicide prevention workforce’, although in some it was included in the broader category of ‘mental health workforce’. Over one third of documents include ‘peer/lived experience workforce’ in paid, voluntary or advocacy positions, in suicide prevention or in a broader mental health context.

The suicide prevention workforce covers diverse settings, including clinical (e.g., emergency department staff, mental health specialists) and non-clinical frontline staff (e.g., paramedics, police), and staff providing ongoing management and care (e.g., GPs, mental health/allied health professionals). Coronial and justice staff, media, teaching staff, and community support professionals can also be included in this category. The multidisciplinary suicide prevention workforce may reflect the whole-of-government approach to suicide prevention and activities to ensure provision of services and supports across a wide range of portfolios and settings.

In terms of capabilities, the *Suicide Prevention Workforce Development and Training Plan for Tasmania* [57] specifies skills required for various workforce categories. Documents reference several existing and new training programs and other initiatives targeting the suicide prevention workforce, including peer workforce, across a range of settings at both the national and state and territory level, including funding for a Centre for Mental Health Workforce Development in Victoria. The Emerging Minds: National Workforce Centre for Child Mental Health aims to build the capacity of mental health workforce focused on children aged 0–12 years and their families.

Consistent with the *National Suicide Prevention Adviser’s Final Advice* [7–10], under the *National Agreement* [26], work is underway involving collaboration between parties to develop the new National Suicide Prevention Workforce Strategy in 2024. This strategy will include workforces and settings where individuals at risk of suicide may present, such as personnel from government departments, service providers, social services, employer groups, community-based organisations, and educators. The *National Lived Experience Workforce Guidelines* [58] aim to create role delineations providing opportunities for contact with consumers, carers, and grassroots advocacy, as well as identifying anti-stigma interventions.

The documents included in the scan also refer to provision of support and retention of skilled and compassionate suicide prevention workforce, including supporting the mental health of health professionals, peer workers, volunteers, and remote suicide prevention workforces.

The NSPLSP offers essential sector leadership and resources particularly through six projects funded to contribute to knowledge gain, exchange, and translation, and build capacity of the suicide prevention sector. In addition, seven of the 18 direct service delivery projects funded via the NSPLSP involve peer-delivered services, ranging from support delivered by youth in educational or (rural) community settings, men in relationship distress, veterans, to peers in Pop-up Safe Spaces.

## Discussion

### Summary of findings

This study aimed to describe government-led suicide prevention activity in Australia and assess the extent of activity alignment with the focus area and enabler components of the NSPO framework underpinning the new Strategy. Both policy documents (plans, strategies, and agreements) and services and programs were considered. Australia’s suicide prevention efforts are significant as demonstrated by activities ranging from policy documents intended to guide and plan activity, the *National Mental Health and Suicide Prevention Agreement* [26] committing the Federal Government and jurisdictions to work together, and the availability of national, state, PHN-region based and digital services and programs. However, we identified only partial or inconsistent progress across each focus area and enabler.

### Implications for suicide prevention policy, practice, and evidence-base

Government-led/funded suicide prevention approaches in Australia are mostly selective or indicated. There is less emphasis on universal approaches, wellbeing promotion, strengthening protective factors and mitigating the impact of known drivers of distress – which require more sustained attention going forward [56]. In addition, there is limited evidence to demonstrate a whole-of-government or whole-of-system approach is operating in Australia. Neither of these findings is surprising given that suicide prevention in Australia is in a period of transition following the *National Suicide Prevention Adviser’s Final Advice* [7–10], which is still relatively recent, and the system will take time to reorient.

Consequently, there are numerous opportunities to improve Australia’s government-led suicide prevention activity. Improving cross-portfolio and cross-jurisdiction collaboration and coordination is relevant to all these opportunities. Importantly, Australia’s suicide prevention policy and practice efforts are not based on a shared framework of suicide prevention, which is crucial to guide collective progress. A shared understanding might be based on the blueprint paper *Understanding Suicide and Self-harm* [12, 13] and the forthcoming new Strategy, both of which consider the role of social determinants and individual-level risk factors and favour a whole-of-government approach that addresses diverse drivers of distress.

There is potential to improve progress across the focus areas outlined in the NSPO framework by drawing on the evidence for social determinants of population health wellbeing, which requires a cross sectoral systems-based approach. Conceptual models that aim to promote such an approach could be applied such as a Canadian framework emphasising the role of collective learning [59] or the Collective Impact Suicide Prevention framework developed in New Zealand [60] highlighting the need for dynamic leadership and resourcing a supporting (‘backbone’) agency to develop and implement cross-sectoral committees and actions.

At a practical level, strategic social services policy and action should support financial security [61] and stable housing [62] given that socio-economic disadvantage (as indicated by interrelated domains such as employment, income, housing, and education) is associated with increasing risk for suicide and self-harm [63, 64]. For example, Medical-Financial Partnerships (MFPs) in the US, involve collaborations between the health sector and financial services organisations to improve health by reducing patient financial stress [65]. Additionally, there are opportunities to adapt or enhance existing services and programs to improve suicide prevention. For example, modelling has shown that health system reform and training health care professionals to detect and reduce suicide risk has the largest potential to reduce the suicide rate [66]. Furthermore, although digital services and online navigation systems help improve access to services round the clock [67], consumers and service providers need to be aware of their existence and ongoing effort is needed to keep them up to date [40].

There is also potential to better leverage the enablers in the NSPO framework. Cross-sector suicide prevention governance, coordination, and alignment could be improved in Australia. For example, consideration could be given to producing a cross-government suicide prevention workplan, which commits each government portfolio to taking action on suicide and outlines deliverables and timeframes for monitoring progress against commitments, as exemplified by the UK [68]. To better embed lived experience decision making and leadership, policy and service delivery principles could draw on findings from a systematic review showing that stakeholder involvement in developing community-based suicide prevention interventions may improve engagement and create opportunities for people with lived experience of a suicidal crisis to provide input, and the need to evaluate the long‐term outcomes of co‐produced suicide prevention interventions [69].

Suicide prevention efforts, particularly those focussed on social determinants of health, are currently hampered by a lack of evidence for their effectiveness and cost-effectiveness making it difficult for government to know which interventions to invest in [70, 71]. Because suicide is a statistically rare event, many potentially useful suicide prevention interventions cannot be evaluated using gold-standard randomised controlled trials (RCTs), particularly universal interventions that target the whole population [70, 72]. The evidence base can be bolstered by conducting rigorous monitoring, evaluation and research based on well-articulated program logic, shared frameworks of suicide and its prevention, and expected intervention outcomes [72–74]. The Outcomes Framework being developed by the NSPO will help promote a shared understanding of outcomes. Additionally, sector wide opportunities for timely cross linkage surveillance data in the service system (e.g., Suicide and Self-harm Monitoring System established by the AIHW) and research environment (e.g., universities) could be capitalised on to improve understanding of suicide attempts and more upstream indicators of distress and wellbeing [75].

Finally in terms of opportunities to strengthen and integrate the clinical and non-clinical suicide prevention workforce, although various relevant competency frameworks exist [10, 76–79], standalone national suicide prevention workforce strategies are lacking internationally [80]. The NSPO led development of the *Australian Suicide Prevention Workforce Strategy* will help fill this international policy gap.

### Limitations and future research

This study does not provide a comprehensive picture of all suicide prevention activity occurring in Australia. It focused primarily on Federal Government (mental) health portfolio-led suicide prevention programs and services, although state and territory-level strategies, and Federal, state and territory joint initiatives were also included. As a result, for programs and services, there is only partial representation of those occurring in other government jurisdictions and portfolios and the community not-for-profit sector. Also, wellbeing promotion activities outside the suicide prevention sector were not included in the scan.

To provide a more complete picture of suicide prevention activity in Australia, future research should focus on activity occurring in non-health portfolios (e.g., social services, employment, education, justice).

## Conclusions

This study found that current government emphasis on and investment in suicide prevention activity, together with strong commitment to lived experience and cross sectorial collaboration, are substantial and appropriate. It has also identified many opportunities to further progress suicide prevention and response efforts as a nation. Suicide prevention efforts can be enhanced by adopting a shared understanding of suicide, which includes the diverse drivers of suicidal distress, and by improving protective factors and social wellbeing. The blueprint paper, *Understanding Suicide and Self-harm* [12, 13], and the *National Suicide Prevention Strategy* the NSPO is developing contribute to this shared understanding. This, in turn, will have implications for expanding, capacity building of, and integrating the clinical and non-clinical suicide prevention workforce. The development of the *National Suicide Prevention Workforce Strategy* by the NSPO will help to drive these reforms. Furthermore, system wide suicide prevention approaches need governance and leadership structures and mechanisms including lived experience leadership (particularly representing priority groups) to ensure they are coordinated, collaborative and informed by the needs of people with lived experience. Findings from this environmental scan illustrate how three decades of Australian government strategies, agreements, services and programs, and investment have been operationalised. This information is useful for comparing approaches in other countries and for informing government policy and resource allocation elsewhere.

## Data Availability

The datasets used and/or analysed during the current study are available from the corresponding author on reasonable request.

## Appendix 1

### Key documents reviewed

#### Policy documents reviewed for alignment with all elements of NSPO framework

1. National Mental Health and Suicide Prevention Agreement (with Bilateral Schedules for each state/territory) [26].
2. Prevention Compassion Care: National Mental Health and Suicide Prevention Plan (2021) [81].
3. National Suicide Prevention Strategy for Australia’s Health System 2020–2023 (2020) [54].
4. National Mental Health and Wellbeing Pandemic Response Plan (Pandemic Response Plan)(2020) [44].
5. Living is for Everyone (LIFE) Framework (2007) [82].
6. National Aboriginal and Torres Strait Islander Suicide Prevention Strategy (2013) [15].
7. National Strategic Framework for Aboriginal and Torres Strait Islander Peoples’ Mental Health and Social and Emotional Wellbeing 2017–2023 (2017) [83].
8. Defence Mental Health and Wellbeing Strategy 2018–2023 (2017) [18].
9. Veteran Mental Health and Wellbeing Strategy and National Action Plan 2020–2023 (2020) [19].
10. The National Children’s Mental Health and Wellbeing Strategy (2021) [20].
11. National LGBTIQ + Mental Health and Suicide Prevention Strategy 2021–2026 (2021) [16].
12. ACT Regional Mental Health and Suicide Prevention Wellbeing Plan 2019–2024 (2019) [84, 85].
13. Strategic Framework for Suicide Prevention in NSW 2018–2023 (2018) [24].
14. NT Suicide Prevention Strategic Framework 2018–2023 (2018) [86].
15. Every life: The Queensland Suicide Prevention Plan 2019–2029: Phase One (2019) [23].
16. South Australian Suicide Prevention Plan 2017–2021(2018) [25].
17. Tasmanian Suicide Prevention Strategy 2023–2027 (2022) [87].
18. Victorian Suicide Prevention Framework 2016–2025(2016) [88].
19. Western Australian Suicide Prevention Framework 2021–2025 (2020) [89].

#### Additional documents reviewed for alignment with lived experience and workforce elements of NSPO framework

Policy documents

1. Fifth National Mental Health and Suicide Prevention Plan (2017) [28].
2. ACT LifeSpan Integrated Suicide Prevention Framework (no date) [90].
3. Suicide Prevention Workforce Development and Training Plan for Tasmania 2016–2020 (2016) [57].

Inquiries and reviews

1. Productivity Commission Inquiry into Mental Health Report (2020) [42].
2. Royal Commission into Victoria’s Mental Health System (2021) [43, 91–95].
3. Australian Government Response to Contributing Lives, Thriving Communities - Review of Mental Health Programmes and Services (2015) [31].
4. Royal Commission into Defence and Veteran Suicide (2022) [96].
5. Vision 2030: Blueprint for Mental Health and Suicide Prevention (2022) [97].
6. AIHW Submission House of representatives Select Committee on Mental Health and Suicide Prevention (2021) [98].
7. Evaluation and Review of the National Suicide Prevention Leadership and Support Program (NSPLSP) (2021) [35].
8. World Health Organization Towards Evidence-based Suicide Prevention Programs (2010) [99].
9. National Children’s Commissioner - Intentional self-harm and suicidal behaviour in children in Children’s Rights Report (2014) [100].
10. The National Suicide Prevention Trial: Insights and Impact (2021) [53].

#### Joint regional plans

**Table:**

PHN region	Plan title
1. Australian Capital Territory	Australian Capital Territory Mental Health and Suicide Prevention Plan Part A: The Framework [84], Part B: implementation plan, Part C: performance and monitoring plan [85]
2. Central & Eastern Sydney	Mental Health and Suicide Prevention Regional Plan [101]
3. Murrumbidgee	Murrumbidgee Regional Mental Health, Suicide Prevention and Alcohol and Other Drugs Regional Plan 2021-24 [102]
4. Hunter, New England and Central Coast	Mental Health Regional Plan 2020–2025 incorporating suicide prevention [103]
5. Nepean Blue Mountains	Joint Regional Mental Health Suicide Prevention Plan (foundation plan) and Strategic Plan [104]
6. Northern Sydney	Northern Sydney mental health, suicide prevention and alcohol and other drugs regional plan 2021–2026 [105]
7. South Eastern NSW	South Eastern NSW regional mental health and suicide prevention plan 2018–2023 (updated 2021) [106]
8. South Western Sydney	Regional mental health and suicide prevention plan to 2025 [107]
9. Western NSW	Regional mental health and suicide prevention plan 2019–2022 [108]
10. Western Sydney	Western Sydney integrated regional mental health and suicide prevention plan 2020–2022 [109]
11. Northern Territory	Northern Territory Mental Health and Suicide Prevention Foundation Plan 2021–2022 [110]
12. Country South Australia	Mental health and suicide prevention regional plan: A joint foundation plan between Country SA PHN and Country Health SA Local Health Network 2019–2021 [111]
13. Adelaide	Towards wellness plan: Adelaide Metropolitan Integrated Mental Health and Suicide Prevention Plan [112]
14. Brisbane North	Planning for wellbeing 2020–2025 revised, A Regional Plan for North Brisbane and Moreton Bay focusing on mental health, suicide prevention, and alcohol and other drug treatment services [113]
15. Brisbane South	Working together differently Brisbane south mental health, suicide prevention, alcohol and other drug foundation plan 2020–2022 [114]
16. Central Queensland	Central QLD, Wide Bay, Sunshine Coast PHN: Joint Regional Mental Health and Suicide Prevention Plan 2020–2025 [115]
17. Darling Downs and West Moreton	Healthy minds. Healthy lives. Darling Downs and West Moreton Joint Regional Mental Health, Suicide Prevention, and Alcohol and Other Drug Plan 2021–2026 [116]
18. Gold Coast	Planning for a compassionate and connected Gold Coast: A Joint regional plan for mental health, suicide prevention, alcohol and other drugs services: foundational plan 2020–2025 and Implementation report July 2021 [117]
19. Northern Queensland	Joint Regional Wellbeing Plan for Northern Queensland [118]
20. Western Queensland	A five year plan (2021–2026) to improve mental health, suicide prevention and alcohol and other drug treatment services in Western Queensland [119]
21. Tasmania	Rethink 2020 a state plan for mental health in Tasmania 2020–2025 and Rethink 2020 implementation plan [120]
22. South Eastern Melbourne	South Eastern Melbourne’s plan for mental health, suicide prevention and alcohol and other drugs 2020–2025 [121]
23. Western Victoria	Joint regional mental health and suicide prevention foundation plan [122]
24. Eastern Melbourne	Regional integrated mental health, alcohol and other drugs and suicide prevention plan for eastern and north eastern Melbourne 2019–2024 [123]
25. Gippsland	Gippsland mental health and suicide prevention plan: foundational plan 2019–2022 [124]
26. Murray	Together: A regional approach to mental health, alcohol and other drugs and suicide prevention foundation plan [125]
27. North Western Melbourne	Regional Foundation Plan blueprint foundation plan 2020-22 [126]
28. WAPHA – Perth South, Perth North, Country WA PHNs	WA Foundation Plan for Mental Health, Alcohol and Other Drug Services, and Suicide Prevention [127]

Note. At the time of our analysis in November 2022, we found 28 regional plans representing 30 of Australia’s 31 PHN regions. The WA regional plan included three PHN regions; and the North Coast area regional was released in December 2022 and was therefore not included in our analysis

## Appendix 2

### Projects funded under the National Suicide Prevention Leadership and Support Program (NSPLSP)

**Table:**

Organisation	Funded activity	Target group	Level of intervention^a^
**ACTIVITY 1: NATIONAL LEADERSHIP IN SUICIDE PREVENTION**			
Suicide Prevention Australia Ltd	Deliver national leadership in suicide prevention and support the broader sector through building partnerships, informing and increasing awareness, advocating and advising and building capacity.	Suicide prevention sector	N/A
**ACTIVITY 2: NATIONAL LEADERSHIP IN SUICIDE PREVENTION RESEARCH TRANSLATION**			
The University of Melbourne	LIFEWAYS: Translating suicide prevention research into policy and practice, through ongoing strategic partnerships (with 7 other universities and NGOs) and collaboration between researchers and end-users of evidence.	Suicide prevention sector	N/A
LGBTIQ + Health Australia (Formerly National LGBTI Health Alliance)	Use datasets from LGBTIQ + communities to provide essential insight into individual and community experiences of mental ill-health and suicidality.	People who identify as LGBTIQ+	N/A
**ACTIVITY 3: CENTRE OF BEST PRACTICE IN ABORIGINAL AND TORRES STRAIT ISLANDER SUICIDE PREVENTION (CBPATSISP)**			
The University of Western Australia	Continue establishing best practice in Indigenous suicide prevention and provide leadership in policy and in development of suicide and self-harm prevention services to Indigenous communities. Operates a clearing house website including best practice guidance and resources to support commissioning of Indigenous suicide prevention activities. Conferences and webinars to support organisations working with Indigenous peoples.	Indigenous suicide prevention sector and community (clearing house)	N/A
**ACTIVITY 4: NATIONAL SUPPORT FOR LIVED EXPERIENCE OF SUICIDE**			
Roses in the Ocean	Provide national leadership in lived experience, including the provision of strategic policy and advice. Build sustainability and grow capability of the lived experience and suicide prevention workforce.	Suicide prevention sector	N/A
Black Dog Institute	Create a network of lived experience participants to support and contribute to the Regional Suicide Prevention Networks	Professionals and people with lived experience working to prevent suicide	N/A
**ACTIVITY 5: NATIONAL MEDIA AND COMMUNICATIONS STRATEGIES**			
Community Broadcasting Association of Australia Ltd	Deliver mental health and suicide prevention messaging over radio stations nationally and develop other communications materials for groups that may be high risk.	Radio broadcasters reaching the public	Universal Selective
Everymind – Life in Mind	National communication program in the form of a digital portal that seeks to reduce suicidal behaviour and stigma through ensuring that those who have a role in suicide prevention can access and apply current data, research, policies, programs, and best-practice communication principles to their work	Suicide prevention sector	Selective
Everymind – Mindframe	National suicide prevention program that provides comprehensive communication guidelines for safe, effective, and responsible reporting, portrayal and communication of suicide and mental ill-health. Mindframe Plus is an extension of the Mindframe program that has been tailored to meet the specific needs of PHNs.	Public PHNs	Universal Selective
LGBTIQ + Health Australia	Undertake awareness-raising campaign of mental ill-health and enabling bystander intervention and encouraging people to reach out for support.	People who identify as LGBTIQ+	Selective
Mental Health First Aid Australia	Mental Health First Aid^®^ International - Addressing the Stigma of Suicide through Mental Health First Aid. Develop a suite of mental health and suicide prevention education materials that complement mental health training programs.	Public to support at-risk populations	Selective
Orygen – The National Centre of Excellence in Youth Mental Health	Extend the #chatsafe program focusing on safe online communication about suicide and self-harm for youth.	Youth Families Educators PHNs Social media industry	Selective
R U OK? Ltd	Deliver campaign activity to build the confidence and capacity of Australians to connect and have conversations about mental health and build the capacity of individuals to be able to support others who are in distress or who may be struggling.	Public	Universal
**ACTIVITY 6: NATIONAL SUICIDE PREVENTION TRAINING**			
Reach Out Australia Pty Ltd	Deliver evidence-based national digital media campaigns targeting broad and at-risk youth populations aged 14–25 years. https://youtu.be/1lc9o14uX6s	Young people aged 16–25	Selective
Anglican Community Services	3-hour QPR (Question Persuade Refer) training program aimed at preventing suicide in seniors and provide support and care to seniors at heightened risk of suicide.	Health professionals working with older adults	Selective
Headspace National Youth Mental Health Foundation Ltd	Deliver a tailored mental health literacy framework to help university staff identify mental health issues.	University staff working with students	Selective
LGBTIQ + Health Australia	Develop new topical resources drawing on most recent best practice to increase awareness in best practice in working with LGBTIQ + people across mainstream and specialist services.	People who identify as LGBTIQ+	Selective
Mental Health First Aid Australia	Mental Health First Aid^®^ International – Mental Health First Aid Training to Strengthen and Build Community Capacity. Increase the scale and impact of training to enable more people to access the skills and knowledge to support to someone experiencing mental health problems or suicidality.	Public to help at-risk populations	Selective
National Aboriginal Community Controlled Health Organisation (NACCHO)	Deliver the Suicide Story program, a unique suicide prevention education and training program developed by Aboriginal people, for Aboriginal people.	Aboriginal communities	Selective
Roses in the Ocean	Deliver evidence-based training (Access and Equity Project) for people with lived experience of suicide living in regions and within at-risk groups.	People with lived experience of suicide without access to this training through fee for service (e.g., men, veterans, LGBTIQ+, older people, youth)	Selective
Wesley Community Services Ltd	Wesley LifeForce suicide prevention training for Network members and health professionals and other gatekeepers in at-risk populations and communities.	Gatekeepers	Selective
Youth Live4Life	Build on the development of Live4Life, an impact model for improving youth mental health and reducing suicide across rural communities. Peer-led model. Delivers mental health first aid training in schools and community, creates local partnership to lead community conversations about mental health and suicide prevention, promotes young leaders as mental health ambassadors.	Youth in rural communities	Selective
**ACTIVITY 7: NATIONAL SUICIDE PREVENTION SUPPORT FOR AT RISK POPULATIONS AND COMMUNITIES**			
Batyr	Deliver evidence-based, peer-to-peer mental health and suicide prevention activities for young people online, at schools and universities.	Young people	Selective
Black Dog Institute	Build on investment commenced under the National Suicide Prevention Trial to deliver evidence-based suicide prevention support services to PHNs, to increase reach and support of at-risk communities. E.g., guidance and resources, utilising Lived Experience expertise on how Veteran, LGBTIQ + and Aboriginal and Torres Strait Islander at-risk cohorts can be safely and effectively supported.	PHNs and other organisations delivering suicide prevention	N/A
Buddy Up	Provide an expanded support network for members transitioning from the Australian Defence Force that gives a sense of connection, identity, and purpose (through physical fitness, social events and purposeful volunteering) to mitigate conditions that lead to post-service depression and post-traumatic stress disorder.	Ex-serving veterans and first responders and their families	Selective
Jesuit Social Services	Deliver online discussion forums for people bereaved by suicide, with a particular focus on people who identify as LGBTIQ + due to their higher risk of suicidality.	People bereaved by suicide with focus on LGBTIQ+	Selective
LGBTIQ + Health Australia	Deliver a program with specialist peer supporters which enables real-time de-briefing and available support for cases that need escalation for people requiring additional support.	People who identify as LGBTIQ+	Selective
MacKillop Family Services	Support young people at risk of suicide by building the capacity of leadership and staff in Australian schools and communities to deliver the Seasons for Growth (SfG) evidence-based change, loss and grief education programs.	Leadership and staff in Australian communities	Selective
MATES in Construction Australia Ltd	National delivery of the MATES in Construction suicide prevention program through community development programs on sites, and by supporting workers in need through case management and a 24/7 help line targeted at male dominated building and construction industries.	People in building and construction industries	Selective Indicated
Menzies School of Health	Develop an app, co-designed and targeting indigenous youth and using evidence-based practices and approaches.	Indigenous youth	Selective
Ngaanyatjarra Pitjantjatjara Yankunytjatjara Women’s Council Aboriginal Corporation	Expand delivery of culturally-based resources for traditional healers to improve Aboriginal social and emotional wellbeing and early intervention activities that strengthen protective behaviours and address the upstream factors that lead to suicide.	Anangu people of Central Australia and people working in Aboriginal health and related services	Selective
OzHelp Foundation Ltd	Use lived experience knowledge to inform the provision of early intervention and wellbeing support (education and training) programs for hard-to-reach workers in high-risk industries (e.g., building and construction, transport and farming and agricultural industries, and rural and regional areas).	Workplaces Communities across Australia	Selective
Parents Beyond Breakup	Deliver evidence-based male suicide prevention support in the form of peer support groups for Dad in Distress	Men experiencing relationship breakdown	Selective
Roses in the Ocean	Develop peer-led Pop-up Safe Spaces in regional and remote communities providing accessible non-clinical alternatives to emergency departments for people in suicidal distress.	People experiencing suicidal distress in regional and remote communities	Indicated
Rural & Remote Medical Services	Deliver an integrated peer-to-peer suicide prevention program conducted in community sporting clubs, with clinical governance and access to online psychologists. Educating young people, health professionals and community about mental health.	Young people Health professionals Community	Universal Selective
The Men’s Table	Expand The Men’s Table program, a preventive strategy addressing social determinants of suicide. Community led, peer to peer, low-cost preventative men’s mental health initiative. Tables comprise about a dozen men who meet once a month over dinner for peer-to-peer support in familiar social settings, such as a private room in the pub. There are now over 70 Tables across Australia with members enjoying this unique experience.	Men	Selective
Villy Australia	Deliver support to veterans and their families transitioning into their post service community. The Villy App recreates the village around them by creating a safe community that gives purpose through allowing them to help others or to receive help.	Veterans and families	Selective
Wesley Community Services Ltd	Wesley Lifeforce Suicide Prevention Networks in [132] communities at heightened risk of suicide and with at risk populations. Most Network members have lived experience of suicide and Networks have a whole of government, community and population reach. Aim to build resilience and reduce suicide within communities impacted by events that increase the risk of suicide (e.g., fire- and flood-affected areas).	At-risk communities	Selective
You Turn Limited	Enhance the national postvention services of the StandBy Program.	People bereaved by suicide	Selective
Youth Insearch	Pilot development of a youth (aged 18-30) Lived Experience Workforce (*n* = 6) in rural communities in Victoria, NSW and Queensland to reduce suicide risk in at-risk youth. Stepped care intervention involving 1:1 case management, weekend workshops and weekly support groups. Overseen by three social workers who will also provide higher intensity interventions if needed.	Youth in rural communities	Selective

Source: A. Warland, personal communication, 2 November 2022. Details of activities expanded using Department of Health and Aged Care [33] and/or web searches

^a^Universal, target the whole population without necessarily identifying individuals who might be at risk of suicide or self-harm; Selective, target individuals who are not yet thinking about suicide or engaging in self-harm, but who are at-risk because they exhibit risk factors that predispose them to do so in the future. This includes interventions directed at gatekeepers working with at-risk populations. Indicated, target individuals who are already suicidal or self-harming [13]

## Appendix 3

### Characteristics of digital mental health services

**Table:**

Service name (Organisation)	Suicide prevention or postvention specific	24/7 crisis	Focus population	Evaluation
Lifeline 13 11 14 Crisis Service (Lifeline Australia)	Yes	Yes	People experiencing crises or other problems	Lifeline Research Foundation conducts research that informs service delivery. All projects receive input from academic partners and Lifeline’s Lived Experience Advisory Group [128].
13 YARN 13 92 76 (13 YARN & Lifeline Australia)	Yes	Yes	Indigenous people having trouble coping	Not found
1800 RESPECT 1800 737 732 (National Domestic Family and Sexual Violence Counselling Service)	No	Yes	People experiencing violence or abuse	Conducted in 2020. 1800RESPECT is delivering a quality counselling service that is highly regarded by callers, staff, the sector and stakeholders. Service improvements were recommended [129].
All Hours Support Service (On the Line)	Yes	Yes	People with low/medium risk of suicide	Not found
Beyond Blue Support Service 1300 22 46 36 (Beyond Blue)	Yes	Yes	People with mental health problems or their families	Effective, most users took help seeking action, more engaged with a health professional, reduced distress, increased confidence to cope and less hopelessness [130].
BeyondNow (Beyond Blue)	Yes	Yes	People experiencing suicidal thoughts	2017 (V1): 1,672 app users completed an online survey (39 users downloaded app on behalf of someone else and 75 health professionals used the app with clients). High usefulness and ease-of-use ratings reported [131]. 2021 (V2): 668 app users completed online survey, and 28 were interviewed or took part in focus groups. High levels of engagement and satisfaction, perceived to be inclusive and neutral in its handling of gender and sexuality, and culturally appropriate and inclusive of First Nations Peoples and LGBTIQA + peoples. Areas for improvement identified [132].
eheadspace (headspace)	Yes	No (9am-1am)	Young people (aged 12-25) experiencing mental health problems	Currently under evaluation. Satisfaction surveys indicate service users and their families accessing eheadspace web chat services are very positive about their experiences [133].
GriefLine (GriefLine)	No	Yes (Online Moderated Forums) No (Helpline: 8am-8pm Mon-Fri)	People experiencing grief and loss	Not found
iBobbly (Black Dog Institute)	Yes	Yes	Young Indigenous peoples (15+) experiencing feeling sad/low or thinking about self-harm	Initial RCT pilot study included 61 Aboriginal people from the Kimberley, WA [134]. Users reported significantly lower levels of depression and psychological distress. iBobbly was well received by people who tried it and feedback from users was positive.
Kids Helpline 1800 551 800 (Yourtown)	No	Yes	Young people (aged 5-25)	Kids Helpline Annual Satisfaction Survey for 2018/19 revealed high levels of satisfaction among the 978 young people who responded [135].
Mensline Australia 1300 78 99 78 (On the Line)	No	Yes	Men with family/relationship problems	Not found
National Indigenous Postvention Service (NIPS) 1800 805 801 (Thirrili)	Yes	Yes	Indigenous peoples, families or communities affected by suicide/trauma	First three years (2017–2020) of development and implementation found that the service is providing valuable postvention support to individuals and families who have recently experienced a suicide or other traumatic event [136]. Improvements in the way the service is managed and delivered were recommended.
Open Arms Veterans & Families Counselling 1800 011 046 (Open Arms)	Yes	Yes	Veterans and families	Not found
Operation Life (OP Life) (Open Arms)	Yes	Yes	Veterans/others experiencing suicidal thoughts	Not found
Qlife 1800 184 527 (LGBTIQ + Health Australia)	No	No (3pm-12am)	LGBTIQ+	Evaluation conducted in October 2021. Recommended improving data input, and liaising with partner sites to develop an improved practice model that responds to the needs of service users, partner sites, and peer supporters [137]. Not found.
Re-Minder Suicide Safety Plan (On the Line)	Yes	Yes	People experiencing suicidal thoughts	Not found
ReachOut.com (ReachOut Australia)	No	Yes (Online community) No (Online peer chat)	Young people aged 14–25/18–25	Significant reduction in symptoms of depression, anxiety and stress, and suicide risk. Increase in help-seeking behaviour [138].
Suicide Call Back Service 1300 659 467 (On the Line)	Yes	Yes	People aged 15 + affected by suicide	Not found
Support After Suicide Program (Stand By Youturn Limited)	Yes	Yes	People bereaved by suicide	A recent independent evaluation of the program found that StandBy’s Support After Suicide service helps to significantly lower the risk of suicidality, mental health concerns, and social isolation following a loss of a loved one [139]. Not found
Way Back Support Service (Beyond Blue)	Yes	Yes	People who have attempted suicide	Evaluation findings indicate reduced psychological distress and suicidal ideation and improved emotional wellbeing [140].

*RCT* Randomised controlled trial

## Appendix 4

### Online navigation systems

**Table:**

Organisation	Service	Description	Target group	Evaluation
Centre of Best Practice in Aboriginal and Torres Strait Islander Suicide Prevention (CBPATSISP)	CBPATSISP Clearing House	CBPATSISP (described under NSPLSP) promotes evidence-based suicide prevention practice that empowers Indigenous individuals, families and communities and respects their culture. The Clearing House shares promising and best practice programs, services, guidelines, resources and research.	Community members, service providers, PHNs, project workers and researchers	Not found
Department of Health and Ageing	Head to Health	Digital mental health gateway. Helps users find digital mental health (including suicide prevention) services from some of Australia’s most trusted mental health organisations. List 730 + digital resources apps, programs, forums, online therapy, websites, phone, chat and email services. Search function allows users to search resources by topic.	Australian mental health consumers and carers Service providers Health professionals	Partially met its objectives and has potential to be cost effective, needs to be promoted, user experience can be improved [39].
Department of Health and Ageing	National Mental Health Platform 1800 595 212 (trial site launched 2022)	Aims to develop Head to Health into a comprehensive national mental health platform that will provide Australians with greater choice in accessing the treatment and services they need (in any communication modality), and more seamless connections across the broader health and mental health system. Includes optional decision support tool (DST; adapted Link-me) to tailor service recommendations according to the user’s predicted severity for depression and/or anxiety and their priorities. The DST does not specifically assess suicidality.	Australian mental health consumers and carers Service providers Health professionals	University of Melbourne will evaluate implementation of DST – users will be able to contribute to the evaluation through the website soon. Users can currently provide feedback via the site.
eMHPrac and Australian Indigenous Health Infonet	WellMob	Social, emotional and cultural wellbeing online resources developed by and for Aboriginal and Torres Strait Islander People. Listing includes suicide prevention resources.	Frontline health and wellbeing workers	Not found
Everymind	Life in Mind	National communication initiative. Gateway connecting Australian suicide prevention services to each other and the community. Includes a directory of suicide prevention programs and services that are not endorsed by Life in Mind.	People working in the suicide prevention sector, as well as those in mental health, government, business or community groups.	Conducts ongoing evaluations to assess its effectiveness in meeting stakeholder needs and priorities and portal functionality. Outcomes from these evaluations are used to make continuous improvements to the online portal [141].
Healthdirect Australia	healthdirect 1800 022 222	Free, trusted virtual health information and advice 24/7. Includes helpline (known as NURSE-ON-CALL in Victoria) operated by registered nurses, an online symptom checker with advice based on symptoms, and a local service finder.	Australian public	Currently under evaluation [142].
PHNs, LHNs, GPs, specialists and others (different in each PHN region)	HealthPathways	Online health information portal implemented across most PHN regions in Australia. Designed for GPs and other primary health clinicians to provide locally agreed evidence-based information on: • How to assess and manage medical conditions; • How to refer patients to local specialists and services in the timeliest way; and • How to improve care pathways for patients. Aims to help them make the right decisions, together with patients, at the point of care. Originated in NZ and used in UK.	Health professionals in primary care	21 studies have evaluated HealthPathways, demonstrating increased awareness and use and identifying that impacts and outcomes are difficult to measure [40].
Queensland University of Technology, Black Dog Institute, Menzies School of Health Research, University Centre for Rural Health North Coast	e-Mental Health in Practice (eMHPrac)	Training and tools for primary health care sector (GPs, allied health professionals and Aboriginal Health Workers) to improve their use of online mental health therapies for their patients. Includes listing of credible Australian online programs, apps, crisis helplines and information sites (updated bi-annually). Services grouped by crisis services, diagnosis, target group, target setting (e.g., school, workplace), delivery mode (app, online, website)	Health professionals in primary care	Internal quality assurance and government reporting. Not in public domain.
ReachOut	ReachOut – Tools and apps	Lists tools and apps that are free and low-cost, including reviews from mental health professionals and users.	Young people Schools Parents	They collect social impact data showing ReachOut reaches young people from diverse backgrounds and produces positive outcomes [143].
Suicide Prevention Australia (SPA)	Suicide Prevention Accreditation Program^a^	National accreditation program to assure safety, quality and efficacy of Australia’s suicide prevention programs. Services listed in the Best Practice Directory have met or are undertaking formal independent assessment against SPA Standards for Quality Improvement	Communities to find quality suicide prevention programs Programs and services to improve safety and quality of program content and delivery	Not found

*DST* Decision support tool

^a^Previously Suicide Prevention Hub in collaboration with Life in Mind

## Abbreviations

ADF: Australian Defence Force
AIHW: Australian Institute of Health and Welfare
AMHC: Adult Mental Health Centre
CALD: Culturally and Linguistically Diverse
CBATSISP: Centre of Best Practice in Aboriginal and Torres Strait Islander Suicide Prevention
DBI: Distress Brief Intervention Trial
GP: General practitioner
IAR-DST: Initial Assessment and Referral Decision Support Tool
NSPLSP: National Suicide Prevention and Leadership Support Program
NSPO: National Suicide Prevention Office
LGBTIQ+: Lesbian, Gay, Bisexual, Transgender, Intersex, Queer/Questioning
LHN: Local Hospital Network
MFP: Medical-Financial Partnership
MBS: Medicare Benefits Schedule
PHN: Primary Health Network

## Footnotes

1 The Federal Government refers to aftercare services for *all* people who have made a suicide attempt as the ‘Universal aftercare services’ initiative. However, according to the public health framework of suicide prevention, ‘universal’ interventions target the whole population; ‘selective’ interventions, people who are at-risk of suicidal ideation or behaviours; and ‘indicated’ interventions, people who are already suicidal or self-harming. Therefore, consistent with this framework, aftercare services – even if they target all people who have made a suicide attempt – are an indicated intervention [13].

## References

[1] World Health Organization. Suicide worldwide in 2019. Geneva: WHO; 2019.

[2] van der Feltz-Cornelis CM, Sarchiapone M, Postuvan V, Volker D, Roskar S, Grum AT, et al. Best Pract Elem Multilevel Suicide Prev Strategies Crisis. 2011;32(6):319–33. 10.1027/0227-5910/a000109 PMC3306243 21945840

[3] Mann JJ, Michel CA, Auerbach RP. Improving suicide Prevention through evidence-based strategies: a systematic review. Am J Psychiatry. 2021;178(7):611–24. 33596680 10.1176/appi.ajp.2020.20060864 PMC9092896

[4] World Health Organization. Live life: an implementation guide for suicide prevention in countries. Geneva: WHO. Licence: CC BY-NC-SA 3.0 IGO; 2021.

[5] Australian Bureau of Statistics. Causes of Death, Australia Canberra: ABS; [https://www.abs.gov.au/statistics/health/causes-death/causes-death-australia/latest-release].

[6] Australian Institute of Health and Welfare. The health impact of suicide and self-inflicted injuries in Australia, 2019 Canberra: AIHW; 2021. [https://www.aihw.gov.au/reports/burden-of-disease/health-impact-suicide-self-inflicted-injuries-2019/contents/summary].

[7] National Suicide Prevention Adviser. Executive Summary. Canberra: Australian Government; 2020.

[8] National Suicide Prevention Adviser. Compassion First: Designing our National Approach from the lived experience of suicidal Behaviour. Canberra: Australian Government; 2020.

[9] National Suicide Prevention Adviser. Connected and compassionate: implementing a National Whole of governments Approach to suicide Prevention. Canberra: Australian Government; 2020.

[10] National Suicide Prevention Adviser. Shifting the focus: supporting a Comprehensive Whole of governments Approach to suicide Prevention. Canberra: Australian Government; 2020.

[11] National Mental Health Commission. About the National Suicide Prevention Office Australia: Australian Government. 2022. [https://www.mentalhealthcommission.gov.au/national-suicide-prevention-office/about-the-national-suicide-prevention-office].

[12] Pirkis J, Gunnell D, Hawton K, Hetrick S, Niederkrotenthaler T, Sinyor M, et al. A Public Health, whole-of-Government Approach to national suicide Prevention Strategies. Crisis. 2023;44(2):85–92. 36960842 10.1027/0227-5910/a000902

[13] Pirkis J, Robinson J, Gunnell D, Hawton K, Hetrick S, Niederkrotenthaler T, et al. Understanding suicide and self-harm. Melbourne: Centre for Mental Health, University of Melbourne; 2022.

[14] National Suicide Prevention Office. National suicide Prevention Strategy Scoping Paper. Canberra: Australian Government; 2022.

[15] Department of Health and Ageing. National Aboriginal and Torres Strait Islander suicide Prevention Strategy. Canberra: Australian Government; 2013.

[16] LGBTIQ + Health Australia. Beyond Urgent: National LGBTIQ + Mental health and suicide Prevention Strategy 2021–2026. Sydney: LGBTIQ + Health Australia; 2021.

[17] Mental Health and Suicide Prevention Division. Suicide Prevention and Digital Branch. Canberra: Australian Government; 2022.

[18] Department of Defence. Defence Mental Health and Wellbeing Strategy 2018–2023. Canberra: Australian Government; 2017.

[19] Department of Veterans’ Affairs. Veteran Mental Health and Wellbeing Strategy and National Action Plan 2020–2023. Canberra: Australian Government; 2020.

[20] National Mental Health Commission. The National Children’s Mental Health and Wellbeing Strategy. Sydney: NMHC; 2021.

[21] NSW Government and Agency for Clinical Innovation. NSW Health suicide care pathways 2022. [ https://aci.health.nsw.gov.au/networks/mental-health/suicide-care-pathway#evidence].

[22] Clinical Excellence Queensland MHAaoDB. Suicide Prevention Practice: Queensland Health Guideline October 2021 2021. [https://www.health.qld.gov.au/system-governance/policies-standards/guidelines].

[23] Queensland Mental Health Commission. Every life: the Queensland suicide Prevention Plan 2019–2029: Phase one. Queensland: Queensland Government; 2019.

[24] Mental Health Commission of New South Wales. Strategic Framework for suicide Prevention in NSW 2018–2023. New South Wales: NSW Government; 2018.

[25] SA Health. South Australian suicide Prevention Plan 2017–2021. South Australia: Government of South Australia; 2018.

[26] Commonwealth of Australia. The National Mental Health and Suicide Prevention Agreement Canberra: Australian Government. 2022. [https://federalfinancialrelations.gov.au/agreements/mental-health-suicide-prevention-agreement].

[27] Health and Social Care. Social Research. Evaluation of the distress brief intervention pilot Programme. Scottish Government; 2022.

[28] Department of Health. The Fifth National Mental Health and suicide Prevention Plan. Canberra: Australian Government; 2017.

[29] Integrated Regional Planning Working Group. Joint Regional Planning for Integrated Mental Health and Suicide Prevention Services: a Guide for Local Health networks (LHNs) and primary Health networks (PHNs). Canberra: Australian Government; 2018.

[30] Australian Institute of Health and Welfare. Suicide prevention activities. Australia’s health 2018. Canberra: AIHW; 2018.

[31] Department of Health. Australian government response to contributing lives, Thriving Communities - Review of Mental Health Programmes and services. Canberra: Australian Government; 2015.

[32] National Mental Health Commission. The National Review of Mental Health Programmes and services. Sydney: National Mental Health Commission; 2014.

[33] Department of Health and Aged Care. The National Suicide Prevention Leadership & Support Program: Project Information for Primary Health Networks. Canberra: Australian Government; 2022.

[34] NDIS Quality and Safeguards Commission. Evidence-informed practice guide, July 2023. Canberra: Australian Government; 2023.

[35] KPMG. Evaluation and review of the National Suicide Prevention Leadership and Support Program: final report. Australia: KPMG; 2021.

[36] Currier D, King K, Oostermeijer S, Hall T, Cox A, Page A, et al. National suicide Prevention Trial final evaluation report, V2. Canberra: Australian Government Department of Health and Aged Care; 2022.

[37] KPMG. Analysis of Suicide Prevention Trials Evaluation Findings. Discussion Paper September 2022. Canberra: Department of Health and Aged Care; 2022.

[38] Waid J, Halpin K, Donaldson R. Mental health service navigation: a scoping review of programmatic features and research evidence. Social Work Mental Health. 2021;19(1):60–79. 10.1080/15332985.2020.1870646

[39] Bassilios B, Ftanou M, Machlin A, Mangelsdorf S, Banfield M, Tan A, et al. Independent evaluation of the Head to Health Digital Mental Health Gateway: final report. Melbourne: Centre for Mental Health, University of Melbourne; 2022.

[40] Senanayake S, Abell B, Novick M, Exley H, Dolejs W, Hutchinson K, et al. Impact and outcome evaluation of HealthPathways: a scoping review of published methodologies. J Prim Health Care. 2021;13(3):260–73. 34588110 10.1071/HC21067

[41] Australian Institute of Health and Welfare. Suicide and self-harm monitoring Canberra: AIHW; 2022. [https://www.aihw.gov.au/suicide-self-harm-monitoring].

[42] Productivity Commission. Mental Health, Report no. 95. Canberra: Australian Government; 2020.

[43] State of Victoria. Royal Commission into Victoria’s Mental Health System, final report, Summary and recommendations, Part Paper No. 202, Session 2018-21 (document 1 of 6). Victoria: Victorian Government; 2021.

[44] Australian Government. National Mental Health and Wellbeing Pandemic Response Plan. Australia: Australian Government; 2020.

[45] Beyond, Blue. Early Childhood Australia, headspace. be you. Canberra: Australian Government; 2022. [https://beyou.edu.au/].

[46] Department of Health. Head to Health: Service Model for Head to Health Adult Mental Health Centres and satellites - revised June 2021. Canberra: Australian Government; 2021.

[47] Australian Government. Initial Assessment and Referral Decision Support Tool Canberra: Commonwealth of Australia. 2022. [https://docs.iar-dst.online/en/latest/index.html#].

[48] Nous Group. Independent Evaluation of HeadtoHelp and AMHCs: final evaluation report. Canberra: Australian Government Department of Health; 2022.

[49] Department of Health and Aged Care. Head to Health Kids National Service Model Canberra: Australian Government. 2022. [https://www.health.gov.au/resources/publications/head-to-health-kids-national-service-model?language=en].

[50] Bowersox NW, Jagusch J, Garlick J, Chen JI, Pfeiffer PN. Peer-based interventions targeting suicide prevention: a scoping review. Am J Community Psychol. 2021;68(1–2):232–48. 33720444 10.1002/ajcp.12510 PMC9165581

[51] Schlichthorst M, Ozols I, Reifels L, Morgan A. Lived experience peer support programs for suicide prevention: a systematic scoping review. Int J Mental Health Syst. 2020;14(1):65. 10.1186/s13033-020-00396-1 PMC7425132 32817757

[52] Australian Public Service Commission. APS Mental Health and Suicide Prevention Unit Canberra: Australian Government. 2022. [https://www.apsc.gov.au/working-aps/diversity-and-inclusion/aps-mental-health-and-suicide-prevention-unit].

[53] Black Dog Institute. The national suicide Prevention Trial: insights and impact. New South Wales: Black Dog Institute; 2021.

[54] National Suicide Prevention Project Reference Group. National Suicide Prevention Strategy for Australia’s Health System 2020–2023. Melbourne: Department of Health and Human Services, Victorian Government; 2020.

[55] Australian Government. National Suicide and Self-Harm Monitoring System Canberra: Department of Health and Aged Care. 2021. [https://www.health.gov.au/our-work/national-suicide-and-self-harm-monitoring-system].

[56] Reifels L, Krysinska K, Andriessen K, Ftanou M, Machlin A, McKay S, et al. Suicide Prevention Research Priorities: final report - October 2022. Melbourne: Centre for Mental Health, University of Melbourne; 2022.

[57] Department of Health and Human Services. Suicide Prevention Workforce Development and Training Plan for Tasmania (2016–2020). Tasmania: Tasmanian Government; 2016.

[58] Byrne L, Wang L, Roennfeldt H, Chapman M, Darwin L, Castles C, et al. National lived experience workforce guidelines. Sydney: National Mental Health Commission; 2021.

[59] de Montigny JG, Desjardins S, Bouchard L. The fundamentals of cross-sector collaboration for social change to promote population health. Glob Health Promot. 2019;26(2):41–50. 28805502 10.1177/1757975917714036

[60] Gale MM. Cross-sectoral suicide prevention implementation post-disasters in Canterbury. Aotearoa New Zealand: University of Canterbury; 2022.

[61] Ngamaba KH, Panagioti M, Armitage CJ. How strongly related are health status and subjective well-being? Systematic review and meta-analysis. Eur J Public Health. 2017;27(5):879–85. 28957478 10.1093/eurpub/ckx081

[62] Chambers D, Cantrell A, Preston L, Peasgood T, Paisley S, Clowes M. Systematic review of the evidence on housing interventions for ‘housing-vulnerable’adults and its relationship to wellbeing. Report. Sheffield: University of Sheffield; 2018.

[63] Mathieu S, Treloar A, Hawgood J, Ross V, Kõlves K. The role of unemployment, Financial Hardship, and economic recession on suicidal behaviors and interventions to mitigate their impact: a review. Front Public Health. 2022;10:907052. 35875017 10.3389/fpubh.2022.907052 PMC9298506

[64] Qin P, Syeda S, Canetto SS, Arya V, Liu B, Menon V, et al. Midlife suicide: a systematic review and meta-analysis of socioeconomic, psychiatric and physical health risk factors. J Psychiatr Res. 2022;154:233–41. 35961179 10.1016/j.jpsychires.2022.07.037

[65] Bell ON, Hole MK, Johnson K, Marcil LE, Solomon BS, Schickedanz A. Medical-financial Partnerships: Cross-sector Collaborations between Medical and Financial Services to improve Health. Acad Pediatr. 2020;20(2):166–74. 31618676 10.1016/j.acap.2019.10.001 PMC7331932

[66] Moutier CY. Innovative and timely approaches to suicide Prevention in Medical Education. Acad Psychiatry. 2021;45(3):252–6. 33954924 10.1007/s40596-021-01459-2 PMC8099389

[67] Torok M, Han J, Baker S, Werner-Seidler A, Wong I, Larsen ME, et al. Suicide prevention using self-guided digital interventions: a systematic review and meta-analysis of randomised controlled trials. Lancet Digit Health. 2020;2(1):e25–36. 33328037 10.1016/S2589-7500(19)30199-2

[68] HM Government. Cross-government suicide Prevention Workplan. UK; 2019.

[69] Hanlon CA, McIlroy D, Poole H, Chopra J, Saini P. Evaluating the role and effectiveness of co-produced community-based mental health interventions that aim to reduce suicide among adults: a systematic review. Health Expect. 2023;26(1):64–86. 36377305 10.1111/hex.13661 PMC9854311

[70] Pirkis J. Background. Paper: Suicide prevention research National Mental Health Commission; 2022.

[71] Maple M, Wayland S, Pearce T, Hua P. Services and programs for suicide prevention: an Evidence Check rapid review brokered by the Sax Institute (www.saxinstitute.org.au) for beyond blue. Sydney: Sax Institute; 2018.

[72] Pirkis J, Hawton K. Evaluating suicide prevention approaches. In: Kõlves K, Sisask M, Värnik P, Värnik A, de Leo D, editors. Advancing suicide research. Gottingen: Hogrefe; 2021.

[73] Centre for Social Impact. Measuring the impact of the Australian suicide Prevention Sector. Final report Sydney: UNSW; 2022.

[74] Centre for Social Impact. Measuring the impact of the Australian suicide Prevention Sector: monitoring and evaluation guide. Sydney: UNSW; 2022.

[75] Flego A, Dempster G, Cutler T, Robinson J, Pirkis J. Evaluation of the national suicide and self-harm Monitoring Project and System. Final Report Australian Institute of Health and Welfare; 2022.

[76] Suicide Prevention Australia. Suicide prevention: a competency framework. Sydney: SPA; 2021.

[77] Suicide Prevention Resource Centre. Core Competencies for Suicide Prevention Program Managers [https://sprc.org/core-competencies/].

[78] Health Education England. National Collaborating Centre for Mental Health. Self-harm and Suicide Prevention Competence Framework. 2018.

[79] NHS Education for Scotland. Scotland’s knowledge and Skills Framework for Mental Health Improvement, self-harm and suicide Prevention. Edinburgh, Scotland: NES; 2019.

[80] Reifels L, Bassilios B, Kenny B, Clapperton A, Skehan J, Krysinska K, et al. Evidence brief. Enabler 4: workforce and community capability. Melbourne: Centre for Mental Health, University of Melbourne; 2023.

[81] Department of Health. National Mental Health and suicide Prevention Plan: Prevention, Compassion, Care. Canberra: Commonwealth of Australia; 2021.

[82] Department of Health and Ageing. Living is for everyone (LIFE): a framework for prevention of suicide in Australia Canberra. Australian Government; 2007.

[83] Commonwealth of Australia. National Strategic Framework for Aboriginal and Torres Strait Islander Peoples’ Mental Health and Social and Emotional Wellbeing: 2017–2023. Canberra: Australian Government; 2017.

[84] Capital Health Network. Australian Capital Territory Mental Health and suicide Prevention Plan 2019–2024, Part A: the Framework. Canberra: Capital Health Network; 2019.

[85] Capital Health Network. Australian Capital Territory Mental Health and suicide Prevention Plan 2019–2024, part B: implementation plan, part C: performance and monitoring plan. Canberra: Capital Health Network; 2019.

[86] Department of Health. Northern territory suicide Prevention Strategic Framework 2018–2023. Northern Territory: Northern Territory Government; 2018.

[87] Tasmanian Government. Tasmanian Suicide Prevention Strategy. Compassion and connection. 2023–2027. Mental Health, Alcohol and Drug Directorate Department of Health; 2022.

[88] Department of Health and Human Services. Victorian suicide Prevention Framework 2016-25. Victoria: Victorian Government; 2016.

[89] Government of Western Australia Mental Health Commission. Western Australian suicide Prevention Framework 2021–2025. Western Australia: Government of Western Australia; 2020.

[90] ACT Health. LifeSpan Integrated Suicide Prevention Canberra: ACT Government; N.D. [https://www.health.act.gov.au/about-our-health-system/office-mental-health-and-wellbeing/lifespan].

[91] State of Victoria. Royal Commission into Victoria’s Mental Health System, final report, volume 1: a new approach to mental health and wellbeing in Victoria, Part Paper No.202, Session 2018-21 (document 2 of 6). Victoria: Victoria Government; 2021.

[92] State of Victoria. Royal Commission into Victoria’s Mental Health System, final report, volume 2: collaboration to support good mental health and wellbeing, Part Paper No.202, Session 2018-21 (document 3 of 6). Victoria: Victorian Government; 2021.

[93] State of Victoria. Royal Commission into Victoria’s Mental Health System, final report, volume 3: promoting inclusion and addressing inequities, Part Paper No.202, Session 2018-21 (document 4 of 6). Victoria: Victorian Government; 2021.

[94] State of Victoria. Royal Commission into Victoria’s Mental Health System, Report F. Volume 4: The fundamentals of enduring reform, Part Paper No.202, Session 2018-21 (document 5 of 6). Victoria: Victorian Government; 2021.

[95] State of Victoria, Report F. Transforming the system innovation and implementation, Part Paper No.202, Session 2018-21 (document 6 of 6). Royal Commission into Victoria’s Mental Health System. Volume 5. Victoria Victorian Government; 2021.

[96] Royal Commission into Defence and Veterans Affairs. Interim report. Australia: Commonwealth of Australia; 2022.

[97] National Mental Health Commission. Vision 2030 for mental health and suicide prevention in Australia. Australia: Australian Government; 2022.

[98] Australian Institute of Health and Welfare. Australian Institute of Health and Welfare Submission: House of Representatives Select Committee on Mental Health and suicide Prevention. Canberra: Australian Institute of Health and Welfare; 2021.

[99] World Health Organization. Towards evidence-based suicide prevention programmes. Manila, Philippines: WHO Regional Office for the Western Pacific; 2010.

[100] Australian Human Rights Commisson. Children’s rights Report. Australia: Australian Human Rights Commission; 2014.

[101] Central, Eastern Sydney PHN. Mental Health and suicide Prevention Regional Plan Central and Eastern Sydney. Sydney, NSW: Central and Eastern Sydney PHN; 2019.

[102] Murrumbidgee Primary Health Network. Murrumbidgee mental health, suicide prevention and alcohol and other drugs regional plan 2021–2024. New South Wales: Murrumbidgee Primary Health Network; 2021.

[103] Hunter New England and Central Coast Primary Health Network. Mental Health Regional Plan 2020–2025 incorporating suicide Prevention: a partnership approach to regional mental health and suicide prevention New South. Wales Hunter New England and Central Coast Primary Health Network; 2021.

[104] Nepean Blue Mountains Primary Health Network. Nepean Blue Mountains Local Health District, Wentworth Healthcare. Joint regional mental health and suicide prevention strategic plan 2021–2026. New South Wales: Nepean Blue Mountains Primary Health Network; 2021.

[105] Northern Sydney PHN. Northern Sydney mental health, suicide prevention and alcohol and other drugs regional plan 2021–2026. Sydney: Northern Sydney PHN; 2021.

[106] South Eastern NSWPHN, Southern NSW, Local Health District ISLHD. South Eastern New South Wales Regional Mental Health and Suicide Prevention Plan, Updated February 2021. New South Wales: South Eastern NSW PHN, Southern NSW Local Health District, Illawarra Shoalhaven Local Health District; 2021.

[107] South Western Sydney PHN, NSW Government. South Western Sydney Regional Mental Health and suicide Prevention Plan to 2025. Sydney, NSW: South Western Sydney PHN, NSW Government; 2020.

[108] Western NSWPHN, Western, NSW Regional Mental Health and Suicide Prevention Plan. A joint foundational plan between Western NSW PHN, Western NSW Local Health District, and Far West Local Health District 2019–2022. New South Wales: Western NSW PHN; 2020.

[109] Western Sydney PHN and Western Sydney Local Health District. Western Sydney integrated regional mental health and suicide prevention plan 2020–2022. Sydney, New South Wales: Western Sydney PHN; 2020.

[110] Northern Territory PHN. Northern Territory Mental Health and Suicide Prevention Foundation Plan 2021–2022: a move towards whole of system integration. Northern Territory: Northern Territory PHN; 2021.

[111] SA Health and Country SA PHN. Mental health and suicide prevention regional plan: a joint foundation plan between Country SA PHN and Country Health SA Local Health Network 2019–2021 South Australia. SA Health and Country SA PHN; 2019.

[112] Adelaide PHN. Towards wellness plan: Adelaide Metropolitan Integrated Mental Health and suicide Prevention Plan Adelaide. Adelaide PHN; 2020.

[113] Brisbane North PHN. Planning for wellbeing: a Regional Plan for North Brisbane and Moreton Bay focusing on mental health, suicide prevention, and alcohol and other drug treatment services. Revised 2020-25. Brisbane: Brisbane North PHN; 2020.

[114] Queensland Government MS, Health, Brisbane South PHN. Working together differently Brisbane South mental health, suicide prevention, alcohol and other drug foundation plan 2020–2022. Brisbane: Brisbane South PHN; 2020.

[115] Central QLD, Wide Bay S, Coast PHN. Mental health, suicide prevention and alcohol and other drugs: Joint Regional Plan 2020–2025. Queensland: Central QLD, Wide Bay, Sunshine Coast PHN; 2020.

[116] Darling Downs and West, Moreton PHN, Darling Downs Health and West Moreton Health. Healthy minds, healthy lives: Darling Downs and West Moreton Joint Regional Mental Health, suicide Prevention and Alcohol and other Drug Plan 2021–2026. Queensland: Darling Downs and West Moreton PHN; 2021.

[117] Gold Coast PHN. Gold Coast Health, Queensland Government. Planning for a compassionate and connected Gold Coast: a joint regional plan for mental health, suicide prevention, alcohol and other drugs services: foundational plan 2020–2025 and implementation report July 2021. Queensland: Gold Coast PHN; 2021.

[118] Northern Queensland PHN. Joint Regional Wellbeing Plan for Northern Queensland: Mental health, suicide prevention, and alcohol and other drugs. December 2020 - version 1. Queensland: Northern Queensland PHN; 2020.

[119] Western Queensland PHN. A five year plan (2021–2026) to improve mental health, suicide prevention and alcohol and other drug treatment services in Western Queensland Queensland. Western Queensland PHN; 2021.

[120] PHN Tasmania and Tasmanian Government. Rethink 2020 a state plan for mental health in Tasmania 2020–2025. Tasmania: PHN Tasmania and Tasmanian Government; 2020.

[121] South Eastern Melbourne Primary Health Network. South-Eastern Melbourne’s plan for Mental Health, Suicide Prevention and Alcohol and other drugs 2020–2025. Melbourne, Victoria: South Eastern Melbourne Primary Health Network; 2020.

[122] Western Victoria PHN. Joint Regional Mental Health and suicide. Prevention Plan Victoria Western Victoria PHN; 2021.

[123] Eastern Melbourne PHN. Regional Integrated Mental Health, Alcohol and other drugs, and suicide Prevention Plan for Eastern and North Eastern Melbourne: 2019–2024. Victoria Eastern Melbourne Primary Health Network: Melbourne; 2019.

[124] Gippsland Primary Health Network. Gippsland Mental Health and suicide Prevention Plan: foundational plan 2019–2022. Gippsland, Victoria: Gippsland Primary Health Network; 2019.

[125] Murray Primary Health Network. Together: a regional approach for mental health, alcohol and other drugs and suicide prevention Foundation Plan. Victoria: Murray Primary Health Network; 2020.

[126] PHN North Western Melbourne. Blueprint for better health: a community report on mental health, alcohol and other drugs and suicide prevention in the North Western Melbourne region. Melbourne: Victoria North Western Melbourne Primary Health Network; 2020.

[127] WA Primary Health Alliance. WA Foundation Plan for Mental Health, Alcohol and Other Drug Services, and suicide Prevention: a collaborative deliverable under the Fifth National Mental Health and suicide Prevention Plan. WA: WAPHA; 2021.

[128] Lifeline. Lifeline research and reports NSW: Lifeline; 2024. [https://www.lifeline.org.au/about/our-research/].

[129] Smyth C, Cortis N, Cama E, Giuntoli G, Breckenridge J, Valentine K. Evaluation of 1800RESPECT – final report. Sydney: Social Policy Research Centre, UNSW Sydney; 2020.

[130] Hawkins A, Odgers J, Reeves A, McCoy A. Evaluating telephone and online psychological support and referral. Evaluation J Australasia. 2020;20(3):157–75. 10.1177/1035719X20927146

[131] Melvin G, Gresham D, BeyondNow. Evaluation of a Safety Planning Smartphone Application. Report: August 2017. Melbourne: Centre for Development Psychiatry & Psychology, Monash University; 2017.

[132] Melvin G, Tatnell R, Gordon MS, James L, Hasking P, Pepping CA, et al. BeyondNow Version 2 evaluation final report. Melbourne: Deakin University; 2021.

[133] headspace. eheadspace satisfaction Snapshot Report. October 2023 Melbourne: headspace: National Youth Mental Health Foundation; 2023 [https://headspace.org.au/assets/eheadspace-Satisfaction-Snapshot_Oct2023.pdf].

[134] Tighe J, Shand F, Ridani R, Mackinnon A, De La Mata N, Christensen H. Ibobbly mobile health intervention for suicide prevention in Australian indigenous youth: a pilot randomised controlled trial. BMJ Open. 2017;7(1):e013518. 28132007 10.1136/bmjopen-2016-013518 PMC5278278

[135] Kids Helpline. Kids Helpline 2018–2019 Annual Client Survey, Direct Counselling Support outcomes. June 2019. Brisbane: yourtown; 2019.

[136] Ridoutt L, Cowles C, Noel J, Kelleher K, Stanford D, Williams M, et al. FINAL REPORT: evaluation of the National Indigenous critical response service. Sydney: Human Capital Alliance; 2020.

[137] LGBTIQ + Health Australia. Expressions of Interest Open for an Evaluation Recommendation-Implementation Consultant Sydney: LGBTIQ + Health Australia; 2021. [ https://www.lgbtiqhealth.org.au/eoi_qlife_implementation_consultant_21].

[138] Kahl BL, Miller HM, Cairns K, Giniunas H, Nicholas M. Evaluation of ReachOut.com, an Unstructured Digital Youth Mental Health Intervention: prospective cohort study. JMIR Ment Health. 2020;7(10):e21280. 33055066 10.2196/21280 PMC7596653

[139] StandBy Support After Suicide. Who we are Queensland: StandBy. 2023. [https://standbysupport.com.au/who-we-are/].

[140] Nous Group. The way back support services evaluation: final evaluation report. Beyond Blue. 21 December 2022. Sydney: Nous Group; 2022.

[141] Life in Mind. Consultations and evaluations NSW: Life in Mind. 2024. [https://lifeinmind.org.au/about-us/consultations-and-evaluations].

[142] UNSW Sydney. Mapping the outcomes of calls to. ‘healthdirect Australia’ Sydney: UNSW; 2024. [https://research.unsw.edu.au/projects/mapping-outcomes-calls-healthdirect-australia].

[143] ReachOut A. Our Impact: 2022–2023 Social Impact Report NSW: ReachOut Australia; [https://about.au.reachout.com/our-impact].

